# Synthesis and characterization of Ce_0.5_Bi_0.5_VO_4_/rGO nanocomposite by sonochemical method for photocatalytic desulfurization of petroleum derivatives

**DOI:** 10.1038/s41598-023-41387-9

**Published:** 2023-08-29

**Authors:** Mohadeseh Farahani, Mehdi Mousavi-Kamazani, Zohreh Salarvand

**Affiliations:** 1 Department of Nanotechnology, Faculty of New Sciences and Technologies, Semnan University, Semnan, Iran; 2 Department of Chemistry, Chemistry and Petrochemistry Research Center, Institute of Standard and Industrial Research of Iran (ISIRI), Karaj, 3174734563 Iran

**Keywords:** Chemistry, Materials science, Nanoscience and technology

## Abstract

In order to improve the desulfurization efficiency of petroleum derivatives, Ce_0.5_Bi_0.5_VO_4_/rGO nanocomposite was synthesized by sonochemical method. The prepared nanocomposites were characterized by XRD, FESEM, EDS, FT-IR, BET, and DRS analyses. XRD analysis shows that the synthesized nanocomposite is amorphous. FESEM images showed that nanostructures with a smaller particle size distribution were synthesized under optimal conditions, i.e. controlling the synthesis temperature between 0 and 5 °C. The results of desulfurization showed that nanocomposites containing reduced graphene oxide (rGO) have higher photocatalytic efficiency than pure samples, the main reason of which can be better charge separation in the samples through the π electron in the rGO structure. The highest amount of desulfurization of CeVO_4_/rGO, BiVO_4_/rGO, and Ce_0.5_Bi_0.5_VO_4_/rGO nanocomposites was 95.62, 91.25, and 96.38%, respectively, after exposure to UV light for 40 min. The enhancement of photocatalytic activity of Ce_0.5_Bi_0.5_VO_4_/rGO composite could be attributed to the efficient separation of electron–hole pairs and the inhibition of recombination. Desulfurization in the presence of hydrochloric acid and hydrogen peroxide increased the efficiency by 12%, which is a significant amount.

## Introduction

In recent decades, critical environmental issues caused by fossil fuels due to the increase in the use of diesel and gasoline fuels and the combustion of sulfur-containing fuels that cause the release of SO_X_^1–3^. It is worth noting that there are different types of sulfur compounds in fuel that release SO_X_ after combustion^4^. Sulfur compounds are toxic and with the rapid development of the automobile industry, they poison the oxidation catalysts that release engine exhaust gases^5^. Sulfur compounds are converted into oxide, sulfate, and sulfur, which causes the production of acid rain, photochemical fog, respiratory problems, and seriously threatens human health and the ecosystem^6,7^.

The process of deep desulfurization of hydrocarbon fuels has been considered due to the requirements of transportation and also due to the effects on the environment. To solve this problem, most countries have developed strict standards to limit the amount of sulfur in fuel. According to these standards, the amount of sulfur is less than 10 ppm and even zero in the future. As a result, one of the most important goals of researchers in recent years is the sweetening of petroleum products from these compounds^8–11^. Therefore, different methods of desulfurization emerged and the most important ones is hydrogen desulfurization. In this process, desulfurization is done by hydrogen under high temperature and pressure with a catalyst^12,13^. To achieve mild operating conditions, other methods were investigated, such as: extractive desulfurization, biological desulfurization, absorption desulfurization, oxidative desulfurization, etc.^14–16^. Photocatalytic oxidative desulfurization is basically an advanced technology of the oxidative desulfurization method that uses an efficient catalyst in the presence of light to increase the oxidation rate of sulfur compounds^17^. This method can be applied at ambient temperature and atmospheric pressure with high selectivity, and due to its low cost and ability to use the sunlight source, it can also be used on an industrial scale^1^. In this method, the electron–hole pair is absorbed on the surface of the catalyst, and this absorption is due to energy higher than or equal to the bandgap that semiconductors can produce hydroxyl holes under ultraviolet light irradiation. Meanwhile, the electron transfers of peroxide to oxygen or peroxide to hydrogen to produce anion, O_2_^**⋅**−^ or hydroxyl radical (**⋅**OH) are strong. The ability to oxidize the basic state on the surface of the catalyst turns it into sulfone, sulfoxide or sulfate ions, which are removed by water^18^.

Types of new photocatalytic materials include materials based on sulfide, nitride, metal oxides, and bismuth. Bismuth-based photocatalysts have received more attention due to their high functionality^19,20^. BiVO_4_ is one of the materials based on bismuth, which, in addition to good performance in absorption, has a small bandgap and high photochemical stability, and is also non-toxic. It has been proven that the photocatalytic activity of bismuth vanadate can be enhanced by other compounds^17,21,22^. One of the compounds that has worked well in coupling with BiVO_4_ for photocatalytic processes is CeVO_4_ because it has a similar structure to bismuth vanadate and thus, the possibility of heterostructure formation is provided. Also, the formation of ⋅O_2_^−^ species that are required for photocatalysis is much easier due to the reaction of adsorbed O_2_ on the surface of CeVO_4_ with Ce^3+^ and e^−^^22,23^. Lu et al.^21^ synthesized BiVO_4_-CeVO_4_ heterojunctions by hydrothermal method for photocatalytic degradation of organic pollutants. The degradation efficiency of levofloxacin in the presence of BiVO_4_ and CeVO_4_ photocatalysts under visible light irradiation was 19.6% and 64%, respectively, while the BiVO_4_-CeVO_4_ heterostructure showed an efficiency of 95.7%. This result confirms that a heterogeneous junction is probably formed between the interface of BiVO_4_ and CeVO_4_ to increase the separation efficiency of the produced carriers. Phadi et al.^24^ synthesized the ternary CeVO_4_/BiVO_4_/rGO nanocomposite for the first time through the hydrothermal method and used it to decompose methyl orange under visible light irradiation. Their results showed that by adding CeVO_4_ to BiVO_4_/rGO composite, the degradation efficiency increases from 57 to 90% after 120 min. Reduced graphene oxide (rGO) is one of the most important compounds that has had a significant effect on improving the activity of photocatalysts, because it has high surface-active sites, high light absorption, and has a high ability to separate the charge between the intrinsic delocalized π–π electrons. Phanichphant et al.^25^ synthesized BiVO_4_ and then combined it with rGO to make a composite through a hydrothermal method. According to their results, BiVO_4_/rGO degraded 90% of methylene blue after 120 min under visible irradiation, while the photocatalytic efficiency of pure BiVO_4_ was about 60%.

In this research, CeVO_4_/rGO, BiVO_4_/rGO, and Ce_0.5_Bi_0.5_VO_4_/rGO have been synthesized through a simple sonochemical method using hydrazine as a reducing agent of GO to rGO. Also, the effect of synthesis temperature on the microstructure and performance of the products has been investigated. The physical and chemical characteristics of obtained materials have been studied by various techniques including XRD, EDS, FESEM, FTIR, and DRS. The performance of the synthesized products in the process of photocatalytic desulfurization of benzothiophene under ultraviolet light irradiation has been investigated and compared. To our knowledge, there is no report on the use of CeVO_4_/BiVO_4_/rGO nanocomposite for photocatalytic oxidative desulfurization.

## Experimental

### Materials and instruments

All materials utilized in this study including cerium(III) nitrate hexahydrate (Ce(NO_3_)_3_⋅6H_2_O), ammonium monovanadate (NH_4_VO_3_), bismuth(III) nitrate (Bi_5_H_9_N_4_O_22_), graphite, hydrazine hydrate (N_2_H_4_⋅H_2_O) (80%), potassium permanganate (KMnO_4_), hydrogen peroxide (H_2_O_2_), hydrochloric acid (HCl), sulfuric acid (H_2_SO_4_), normal hexane (C_6_H_14_), and benzothiophene (BT) were purchased from Merck and Sigma-Aldrich companies, and used as-received with no further purification. Ultrasound was performed using an ultrasonic 12 mm diameter probe, operating at 20 kHz with an output power of 400 W cm^−2^ optimized with a calorimeter. XRD (X-ray Diffraction) patterns were analyzed by a Philips-X'PertPro device using Ni-filtered Cu Kα radiation. A Zeiss sigma300-HV device was used to record FESEM (field-emission scanning electron microscope) images. Fourier transform infrared (FT-IR) analysis was performed with a Magna-IR device, a Nicolet 550 spectrometer with a resolution of 0.125 cm^−1^ in KBr tablets in the range of 400 to 4000 cm^−1^. EDS (energy dispersive spectroscopy) analysis was performed using a Philips XL30 x-ray scattering device. Reflectance spectrometry (DRS) analysis was performed by Shimadzu model UV3600Iplus. N_2_ adsorption/desorption (BET) analysis was performed by Belsorp mini x device. To measure the amount of sulfur, a sulfur analyzer in oil model Horiba-SLFA-20 was used.

### Synthesis of CeVO_4_/BiVO_4_/rGO nanocomposite

**Figure 1:**
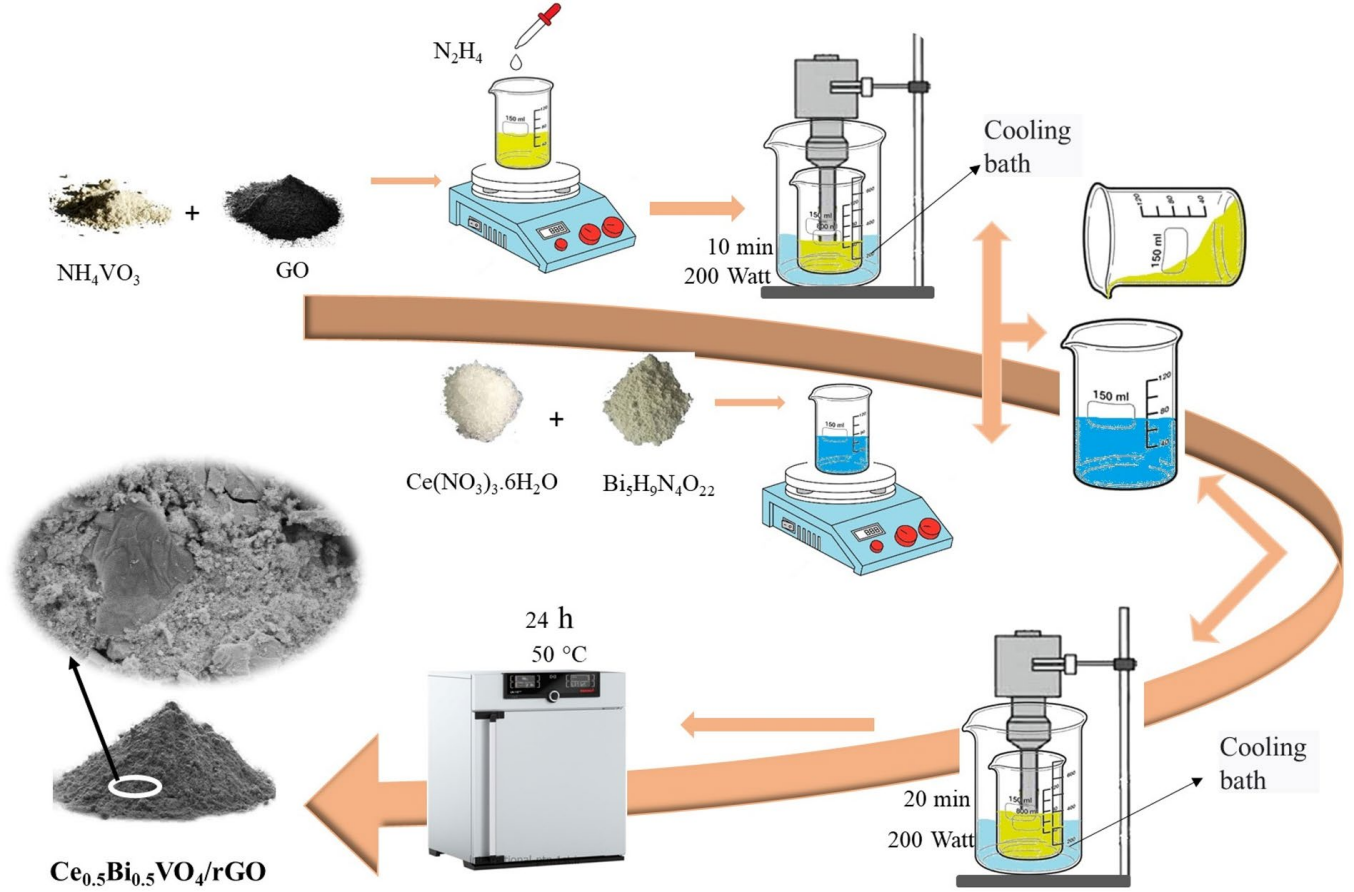
Synthesis steps of Ce_0.5_Bi_0.5_VO_4_/rGO nanocomposite.

Graphene oxide (GO) was synthesized by Hummer's advanced method^26^. First, 1 mmol of ammonium vanadate (0.116 g) and 0.05 g of graphene oxide were poured into 50 ml of distilled water and placed on a stirrer for 20 min. Then, 3 ml of hydrazine was added and stirring was continued for 5 min. The carrier solution was irradiated for 10 min under ultrasonic waves with a power of 200 W. In this step, by adding hydrazine, graphene oxide was reduced to rGO. 0.5 mmol of cerium nitrate and 0.5 mmol of bismuth nitrate were dissolved in 30 ml of distilled water and added to the first solution and irradiated with ultrasound waves with a power of 200 W for 20 min. For the synthesis of CeVO_4_/rGO and BiVO_4_/rGO nanocomposites, 1 mmol of each salt was removed in the second step and the rest of the steps were the same as before. All the above steps were repeated once by controlling the temperature between 0 and 5 °C by a cooling bath in order to investigate the effect of temperature. In the absence of a cooling bath, the temperature of the solution rose up to 55 °C. Figure 1 shows the synthesis steps of Ce_0.5_Bi_0.5_VO_4_/rGO nanocomposite. The reaction conditions for the preparation of CeVO_4_/BiVO_4_/rGO nanocomposite are listed in Table 1.

**Table 1:** The reaction conditions of the preparation of CeVO_4_/BiVO_4_/rGO nanocomposite.

Sample no	Ce (mmole)	Bi (mmole)	GO (g)	Power (W)	Time (min)	Temperature (°C)	Product
S1	1	–	–	200	30	55–60	CeVO_4_
S2	–	1	–	200	30	55–60	BiVO_4_
S3	0.5	0.5	–	200	30	55–60	CeVO_4_/BiVO_4_
S4	1	–	0.05	200	30	55–60	CeVO_4_/rGO
S5	–	1	0.05	200	30	55–60	BiVO_4_/rGO
S6	0.5	0.5	0.05	200	30	55–60	CeVO_4_/BiVO_4_/rGO
S7	1		0.05	200	30	0–5	CeVO_4_/rGO
S8	–	1	0.05	200	30	0–5	BiVO_4_/rGO
S9	0.5	0.5	0.05	200	30	0–5	CeVO_4_/BiVO_4_/rGO
S10	0.5	0.5	0.05	–	–	20–25	CeVO_4_/BiVO_4_/rGO

### Photocatalytic desulfurization of benzothiophene

First, 1000 ml of 800 ppm sulfur solution was prepared from benzothiophene in normal hexane. Then 100 ml of the above 800 ppm standard solution and 0.1 g of photocatalyst powder were poured into the beaker and was placed inside the reactor. In order to establish a balance between adsorption and desorption, the obtained mixture was stirred for 30 min under aeration in the dark on a stirrer and then irradiated with a 400 W Osram UV lamp. The distance between the lamp and the solution was set to 20 cm. A distillation column was used to prevent the evaporation of the solvent. In order to measure sulfur, 15 ml of solution was taken at specific time intervals and then separated by a centrifuge. Then, the solution with 15 ml acetonitrile (BT/CH_3_CN: 1/1) as an extractor was stirred for 5 min and centrifuged with high speed again. After that, the two phases were separated, the upper phase was extracted for determining of the amount of its sulfur content. The amount of sulfur in the samples was measured using a sulfur in oil measuring device. In order to investigate the effect of H_2_O_2_ and HCl, desulfurization of sample S10 was done once in the presence of H_2_O_2_ and HCl.

## Results and discussion

### XRD studies and mechanism of Ce_0.5_Bi_0.5_VO_4_/rGO formation

**Figure 2:**
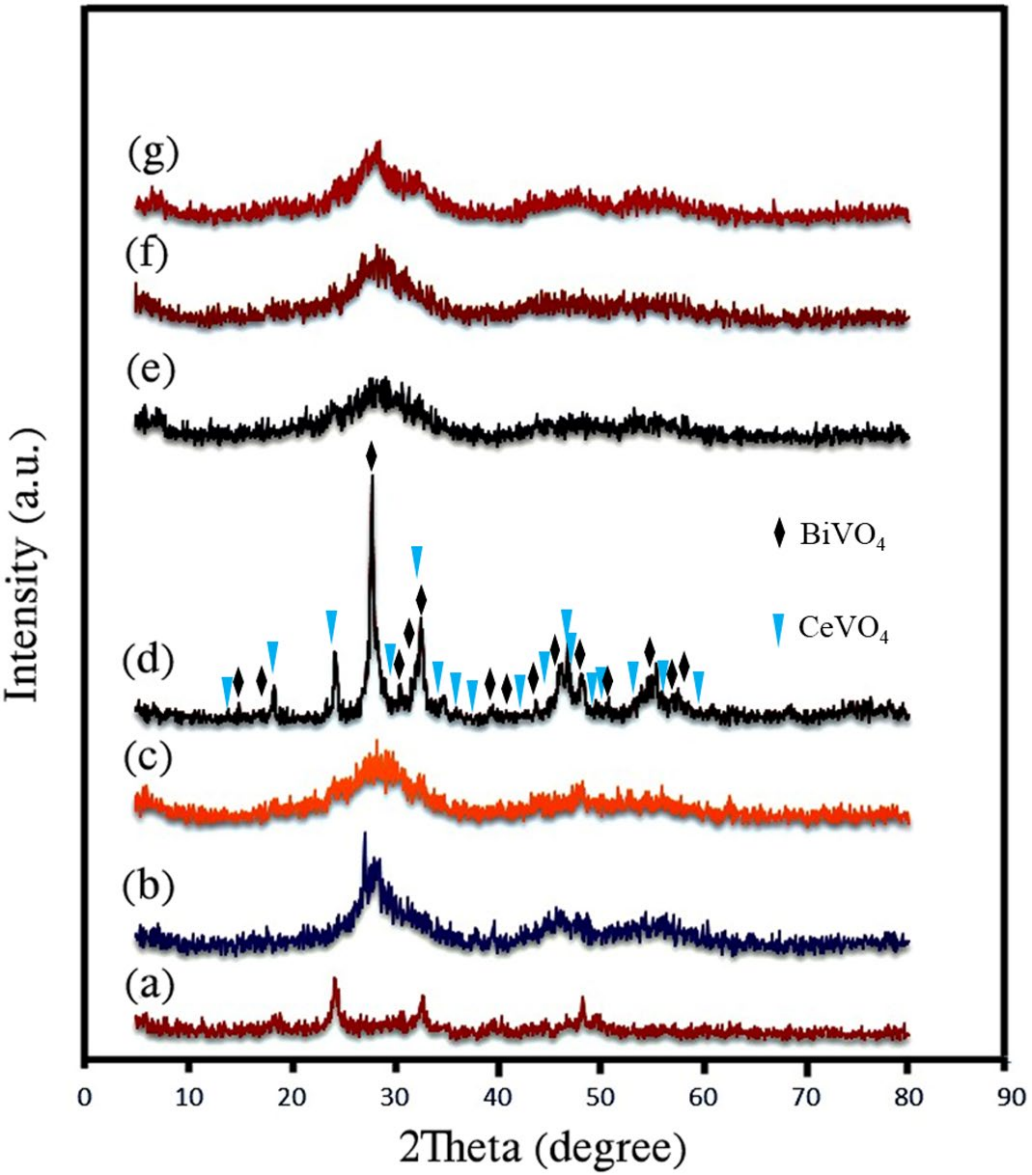
XRD patterns of the as-synthesized products (**a**) sample S1, (**b**) sample S2, (**c**) sample S3, and (**d**) sample S3 after calcination, (**e**) sample S6, (**f**) sample S9, and (**g**) sample S10.

X-ray diffraction patterns of samples S1, S2, and S3, which belong to CeVO_4_, BiVO_4_, and CeVO_4_/BiVO_4_, respectively, are shown in Fig. 2a–c. As can be seen, the samples are amorphous, which is due to the use of ultrasound waves for synthesis. Figure 2d corresponds to sample S3 (CeVO_4_/BiVO_4_) after calcination at 400 °C. According to the patterns in Fig. 2d, some peaks correspond to the tetragonal structure of CeVO_4_ (JCPDS = 01072028 and lattice parameters a = 7.34 Å, b = 7.34 Å, c = 6.47 Å) and the rest of the peaks belong to the tetragonal structure of BiVO_4_ (JCPDS = 000140113, lattice parameters a = 7.2999 Å, b = 7.2999 Å, c = 6.4573 Å). The absence of additional peaks shows that there are no impurities such as cerium oxide, Bi, and Bi_2_O_3_ and confirms the purity of samples 1–3. Figure 2e–g are related to samples S6, S9, and S10, all of which contain CeVO_4_/BiVO_4_/rGO ternary composite synthesized in different conditions. Sample S6 is the synthesized sample without temperature control. Sample S9 is the sample synthesized with temperature control between 0 and 5 °C, and sample S10 is the sample that was synthesized without ultrasonic waves. By examining patterns in Fig. 2a–c and comparing them with Fig. 2d, it can be concluded that samples S6, S9, and S10 are pure and no additional peaks caused by impurities are observed. Due to the presence of carbon in the ternary nanocomposite, none of these samples can be calcined. Despite the amorphous nature of the samples, the X-ray diffraction test was also performed for these samples to ensure their purity. The absence of a peak at about 2θ = 10.6° indicates that the complete graphene oxide has been converted into reduced graphene oxide^27^.

### EDS studies

**Figure 3:**
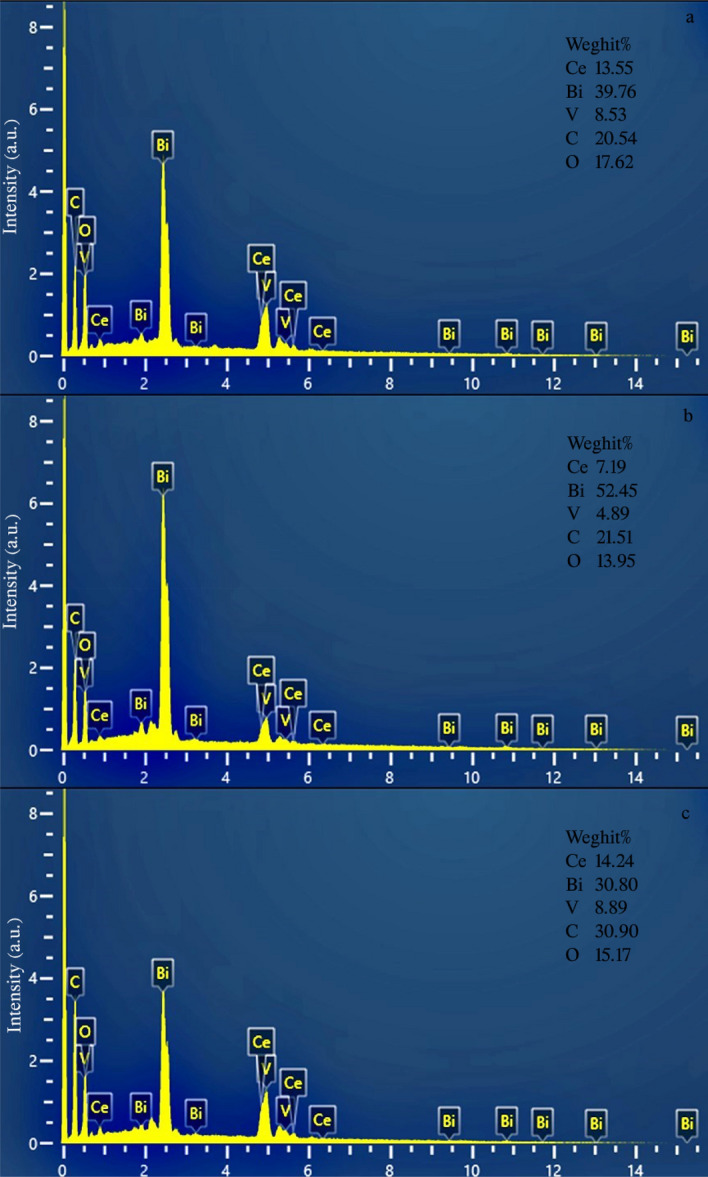
EDS spectra of the as-synthesized products (**a**) sample S6, (**b**) sample S9, and (**c**) sample S10.

Quantitative and qualitative analysis of the chemical composition of nanocomposites using EDS spectroscopy is presented in Fig. 3a–c. Spectra a, b, and c belong to samples S6, S9, and S10, and as it is clear, only Ce, Bi, V, C, and O peaks are seen, which reveals the purity of the products and the absence of impurities. Figure 3b corresponds to the product obtained with temperature control. As can be seen, the weight percentage of bismuth has increased, from which it can be concluded that the conditions for the formation of bismuth vanadate are better at a lower temperature. In Fig. 3a and c, the percentages are almost close to the stoichiometric percentages and no contamination is seen.

### FTIR spectrum

**Figure 4:**
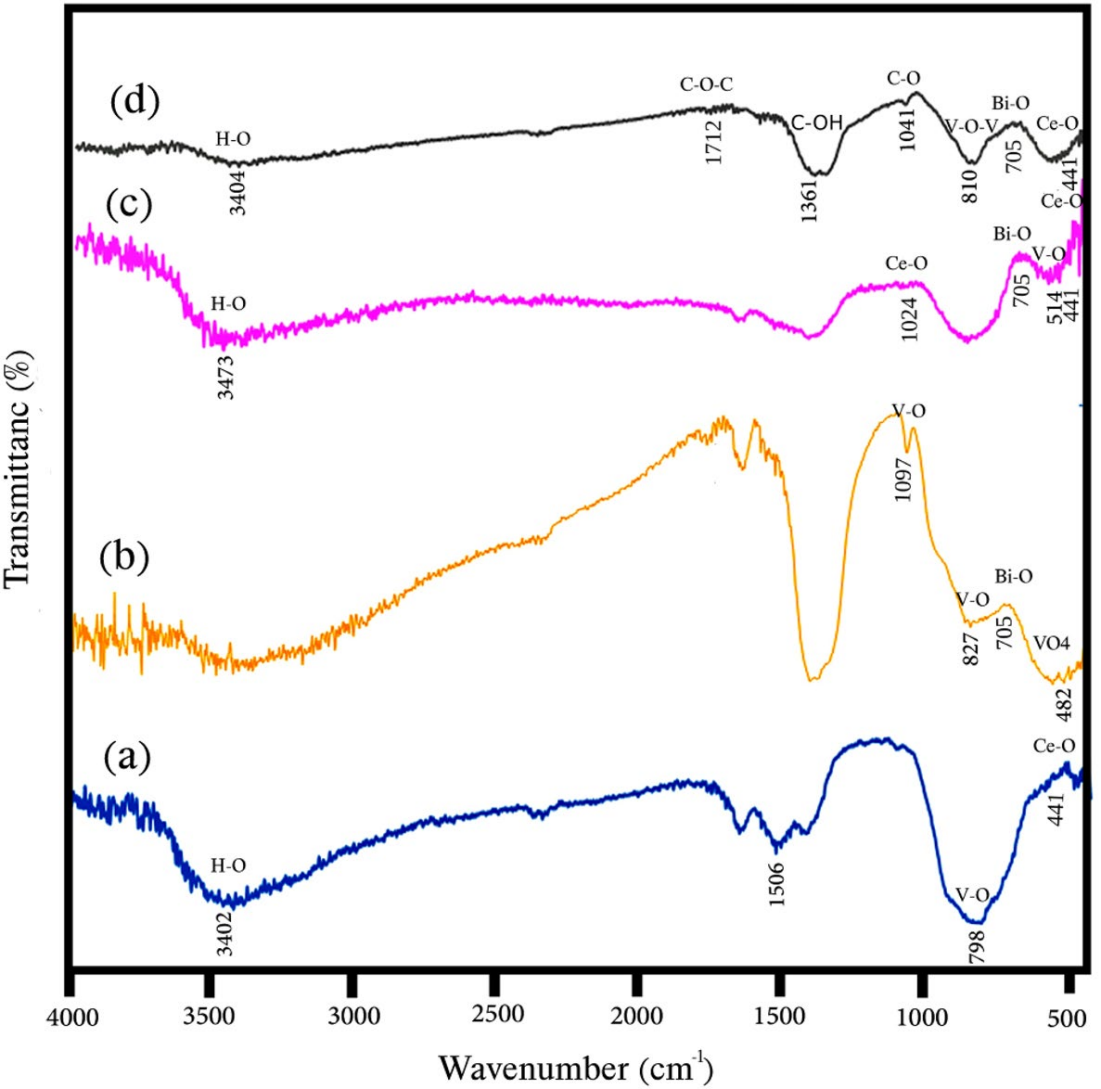
FT-IR spectra of the as-synthesized products (**a**) sample S1, (**b**) sample S2, (**c**) sample S3, and (**d**) sample S6.

The FTIR spectroscopy is used to investigate chemical bonds and organic groups and the results is presented in Fig. 4. In all the spectra, the band around 3400 cm^−1^ is characteristic of the O–H stretching vibration, which indicates the absorbed water. In Fig. 4a, which is related to sample S1 (CeVO_4_), the two bands at 441 cm^−1^ and 798 cm^−1^ show the stretching vibrations in Ce–O and V–O bonds, respectively^28–31^. The band at 1506 cm^−1^ corresponds to the carbonate ion absorbed from the air, which indicates impurity on the surface of the nanoparticle and the reason can be the presence of some hydrazine on the surface of the cerium vanadate nanoparticle^7^. Figure 4b shows the spectrum of sample S2 (BiVO_4_). The band at 482 cm^−1^ belong to the bending vibrations of VO_4_. The 827 cm^−1^ and 1097 cm^−1^ bands are related to V–O vibrations and the 705 m^−1^ band is related to Bi-O vibrations^32–34^. The FT-IR spectrum of sample S3 (Ce_0.5_Bi_0.5_VO_4_) is presented in Fig. 4c, where the stretching bands at 441 cm^−1^ and 1024 cm^−1^ attributed to Ce–O vibrations^25,28^. The stretching bands at 705 cm^−1^ and 514 cm^−1^ corresponds to the Bi-O, and V–O vibrations, respectively^25^. The FTIR spectrum of sample S6 (Ce_0.5_Bi_0.5_VO_4_/rGO) is shown in Fig. 4d, where the bands at 705 cm^−1^ and 441 cm^−1^ correspond to the stretching vibrations of Bi-O and Ce–O, respectively. The band at 810 cm^−1^ is due to V–O and V–O–V vibrations, while the band at 1361 cm^−1^ is related to the transformation of O–H to C–OH, which indicates the conversion of GO to rGO^35^.

### FESEM studies

The following mechanism is proposed for the formation of cerium vanadate^27^:

1 $${\mathrm{Ce}}^{3+}+{3\mathrm{H}}_{2}\mathrm{O}\to {\mathrm{Ce}(\mathrm{OH})}_{3}+{{3\mathrm{H}}^{+}}_{(aq)}$$

2 $${{\mathrm{VO}}_{3}}^{-}+{\mathrm{OH}}^{-}\to {{\mathrm{VO}}_{4}}^{3-}+{\mathrm{H}}^{+}$$

3 $${\mathrm{Ce}(\mathrm{OH})}_{3}+{{\mathrm{VO}}_{4}}^{3-}\to {\mathrm{CeVO}}_{4}+3{\mathrm{OH}}^{-}$$

A mechanism similar to that of cerium vanadate has been proposed for the formation of bismuth vanadate.

**Figure 5:**
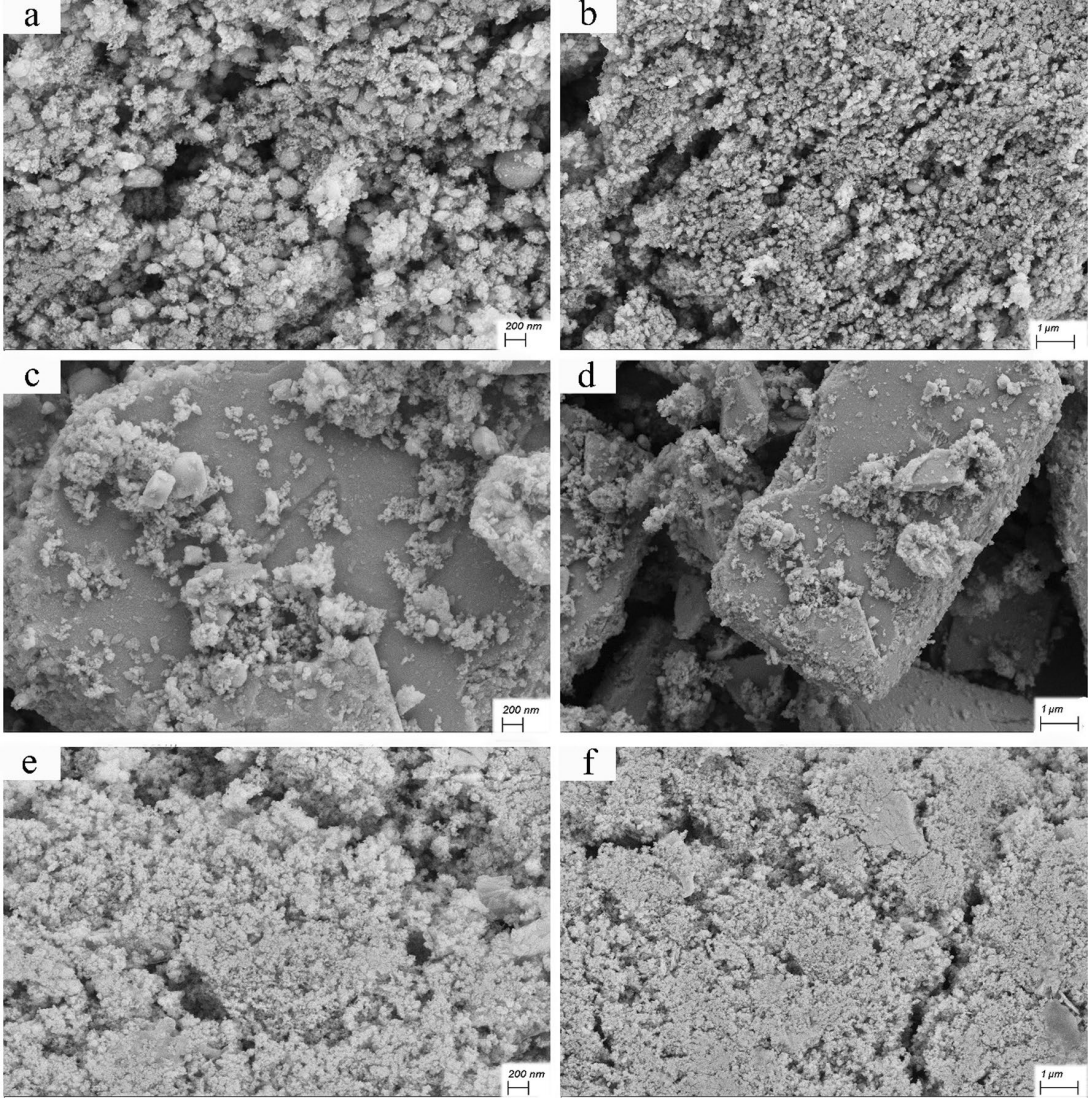
FESEM images of the as-synthesized products (**a**) and (**b**) sample S1, (**c**) and (**d**) sample S2, and (**e**) and (**f**) sample S3.

**Figure 6:**
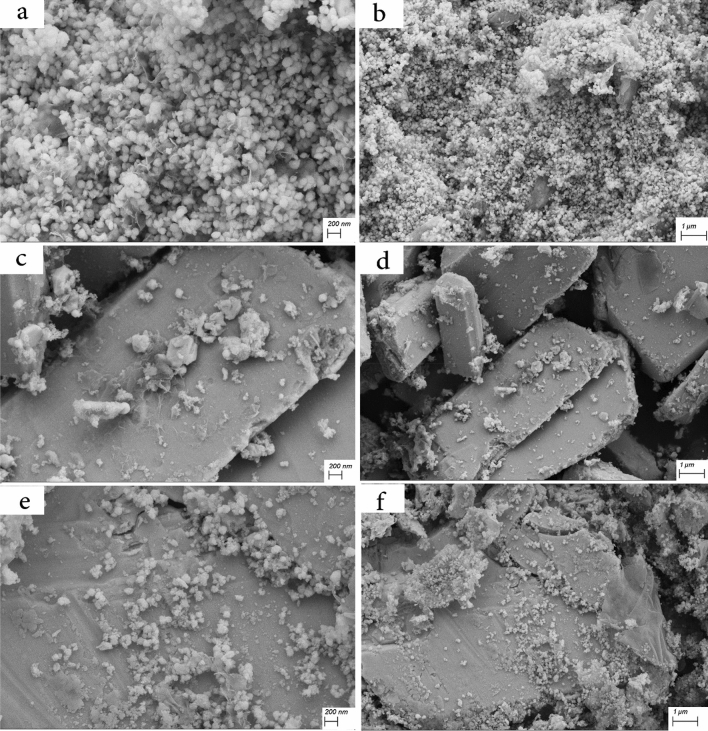
FESEM images of the as-synthesized products (**a**) and (**b**) sample S4, (**c**) and (**d**) sample S5, and (**e**) and (**f**) sample S6.

**Figure 7:**
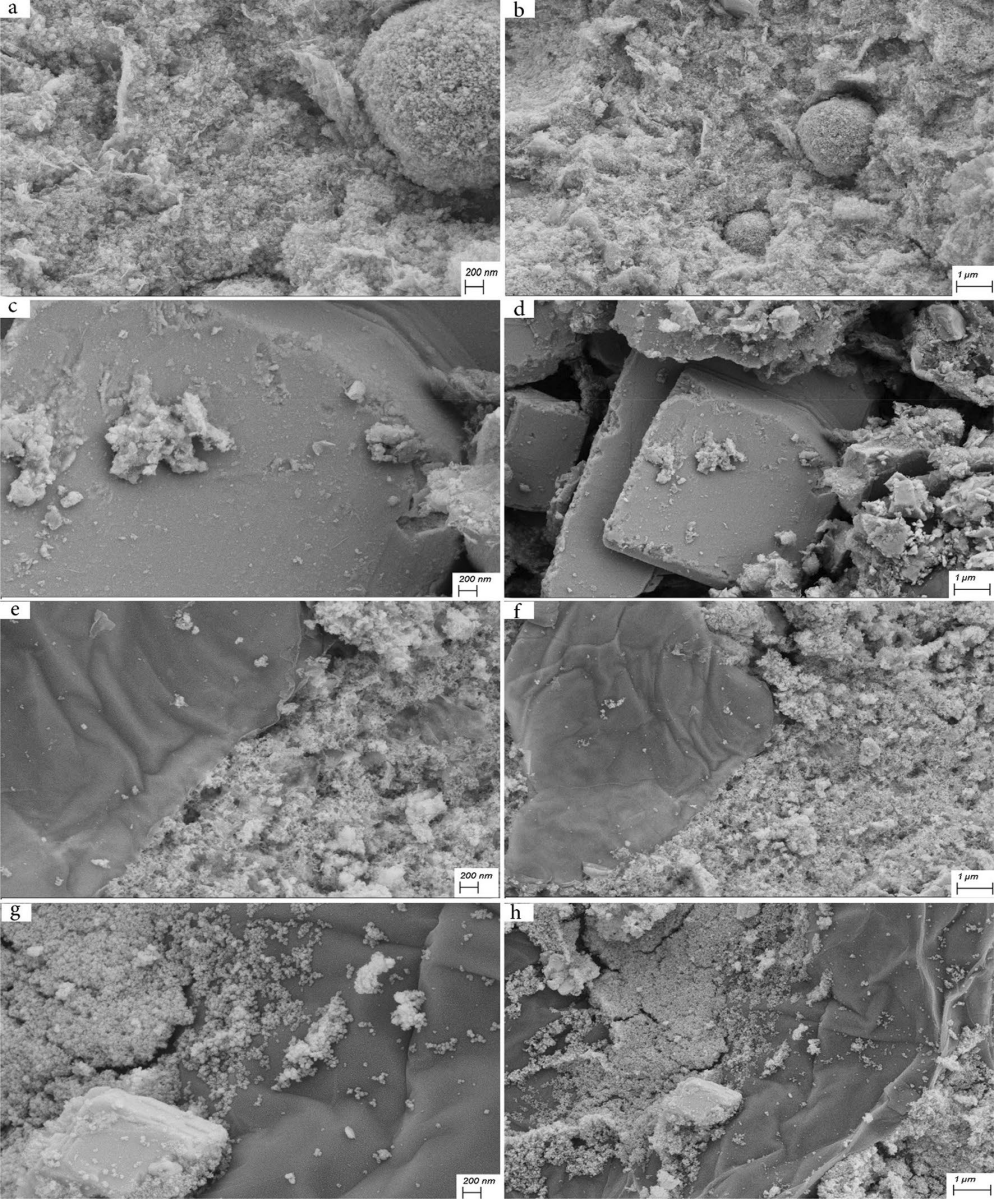
FESEM images of the as-synthesized products (**a**) and (**b**) sample S7, (**c**) and (**d**) sample S8, and (**e**) and (**f**) sample S9, and (**g**) and (**h**) sample S10.

Figure 5 shows the FESEM images of samples S1, S2, and S3 with two different magnifications. As seen in Fig. 5a and b, sample S1 contains CeVO_4_ nanoparticles with a spherical morphology with an approximate diameter of 25–50 nm, which have good uniformity. Figure 5c and d correspond to sample S2 (BiVO_4_). As can be seen, bismuth vanadate nanoparticles with rectangular cube morphology with micrometer size have been synthesized. Figure 5e and f are related to Ce_0.5_Bi_0.5_VO_4_ nanocomposite (sample S3). In Fig. 5e, CeVO_4_ nanoparticles with an average particle size between 15–50 nm can be seen. These nanoparticles are located on a rectangular cube surface, which according to Fig. 5c corresponds to the morphology of bismuth vanadate. According to Fig. 6a and b, which is related to CeVO_4_/rGO nanocomposite, CeVO_4_ nanoparticles and reduced graphene oxide sheets are well visible. The diameter of CeVO_4_ nanoparticles is approximately between 10 and 25 nm and the thickness of graphene sheets is about 10–20 nm. Figure 6c and d are related to sample S5 (BiVO_4_/rGO nanocomposite). According to the pictures, the reduced graphene oxide sheets are placed on the bismuth vanadate surface. But in the case of cerium vanadate, these plates were located between nanoparticles. Bismuth vanadate with rectangular cube morphology and average dimensions between 1 and 5 µm can be seen in Fig. 6c. In Fig. 6d, graphene sheets with a thickness of approximately 10–30 nm are clearly visible on the surface. Figure 6e and f are related to sample S6 (Ce_0.5_Bi_0.5_VO_4_/rGO nanocomposite). In Fig. 6e, CeVO_4_ nanoparticles and reduced graphene oxide sheets are clearly visible, which are unevenly distributed on the surface, and cerium vanadate nanoparticles are placed between the graphene sheets. The size of cerium vanadate nanoparticles is between 10 and 25 nm and the average thickness of graphene sheets is about 8–25 nm. In Fig. 6f, bismuth vanadate, on the surface of which CeVO_4_ nanoparticles and reduced graphene oxide sheets are non-uniformly accumulated, can be clearly seen. FESEM images of CeVO_4_/rGO nanocomposite can be seen in Fig. 7a and b. This sample was synthesized by controlling the temperature between 0 and 5 °C. By examining the effect of temperature on the synthesis of this nanocomposite, it can be concluded that the distribution of CeVO_4_ particles among the reduced graphene oxide sheets was more uniform. CeVO_4_ nanoparticles have been produced with a particle size between 5 and 20 nm, which is smaller and more uniform than when the temperature was not controlled. Reduced graphene oxide sheets have a thickness between 8 and 15 nm. Due to the better distribution and smaller size of the nanoparticles in Fig. 7b, these nanoparticles could not be well detected, while the nanoparticles and graphene oxide sheets were well detected when there was no temperature control in the synthesis. Figure 7c and d show the SEM images of sample S8 (BiVO_4_/rGO nanocomposite). In Fig. 7c, the reduced graphene oxide nanosheets can be clearly seen. The thickness of these plates is between 5 and 10 nm and their distribution on the surface is clearly visible. In Fig. 7d, BiVO_4_, which has a rectangular cube morphology, can be seen with a height of 500–650 nm and different lengths that reach up to 5.5 μm. In this image, similar to Fig. 7b, the graphene sheets are not well visible, which is due to the more uniform distribution and smaller particle size. According to Fig. 7e and f, which corresponds to sample S9 (Ce_0.5_Bi_0.5_VO_4_/rGO nanocomposite), the effects of temperature control are clearly evident. Reduced graphene oxide nanosheets with a thickness of 5–20 nm and CeVO_4_ nanoparticles with a particle size of 15–25 nm can be seen in Fig. 7e. In this image, the particle size distribution among the reduced graphene oxide sheets is more uniform than when there is no temperature control. In Fig. 7f, the accumulation of CeVO_4_ nanoparticles and reduced graphene oxide nanosheets on the BiVO_4_ surface can be seen. In this image, the particle size distribution is more uniform, but because of the magnification of the image, the compositions are not well visible. FESEM images of Ce_0.5_Bi_0.5_VO_4_/rGO nanocomposite synthesized in the absence of ultrasonic waves (Sample S10) are presented in Fig. 7g and h. As seen in Fig. 7, the CeVO_4_ nanoparticles have a size between 25 and 50 nm, and the dispersion range is larger when the synthesis temperature is between 0 and 5 °C, but smaller than when there is no temperature control on the synthesis. The reason for this could be that the synthesis temperature of this nanocomposite is between two other composites and it was done at ambient temperature. In this image, unlike other images, the reduced graphene oxide sheets are not visible, but a part of the rectangular BiVO_4_ microcube is visible. According to Fig. 7h, it is clear that unlike other samples, the reduced graphene oxide sheets have three sides or at least two sides in the nanoscale. In this sample, these sheets are two-dimensional nanosheets and their two dimensions are not at the nanoscale, and unlike the other 5 samples, CeVO_4_ and BiVO_4_ are accumulated on reduced graphene oxide sheets. Figure 7h shows that graphene oxide and cerium vanadate plates are accumulated on the surface of rectangular bismuth vanadate microcubes.

### BET analysis

**Figure 8:**
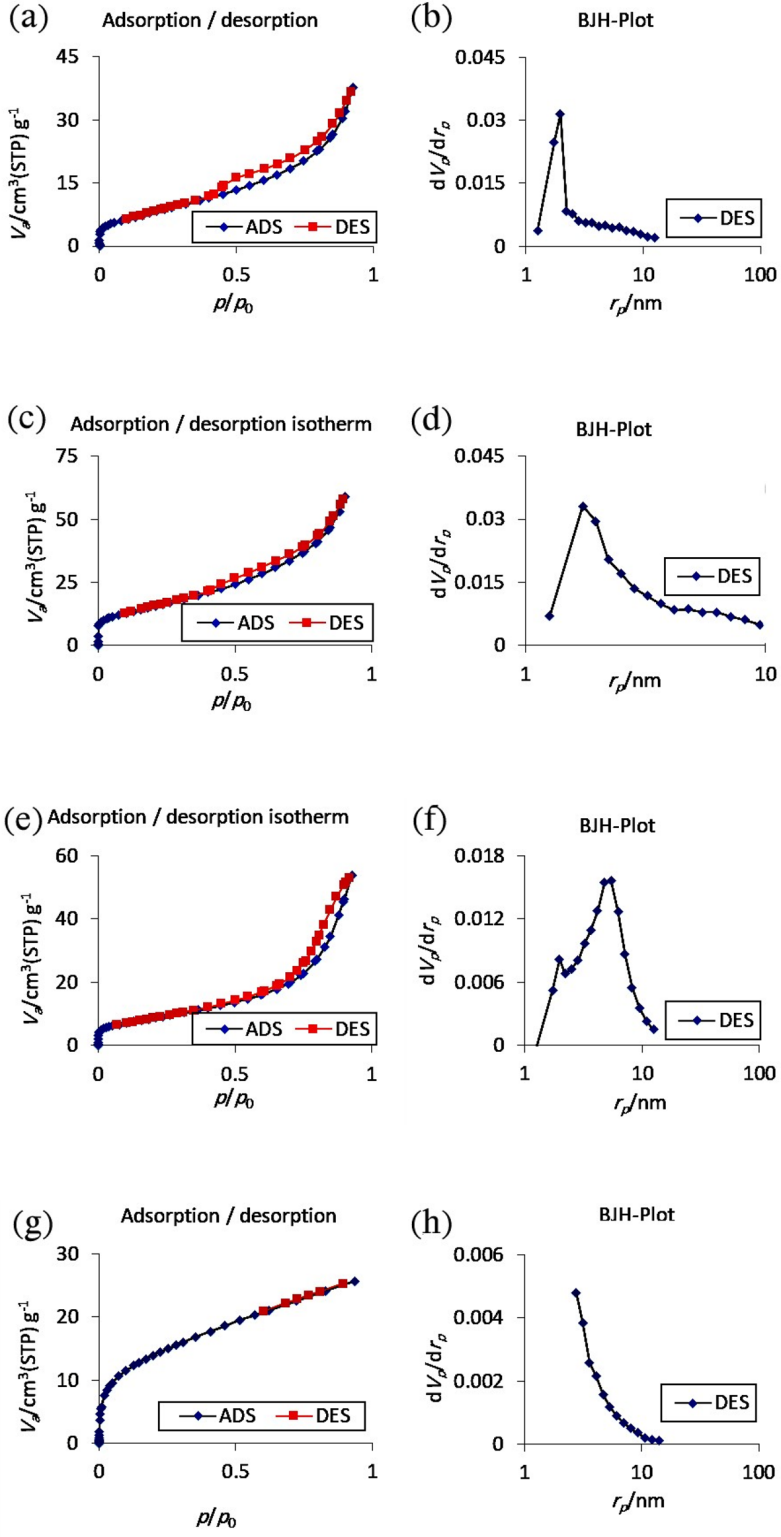
N2 adsorption/desorption isotherms and pore size distribution of BJH (**a**) and (**b**) sample S6, (**c**) and (**d**) sample S9, and (**e**) and (**f**) sample S10, and (**g**) and (**h**) sample S7.

The specific surface area is one of the factors affecting the photocatalytic performance. Therefore, the adsorption–desorption properties as well as the pore size distribution of CeVO_4_/BiVO_4_/rGO nanocomposites have been investigated. Figure 8 shows the adsorption–desorption isotherms and pore size distribution of samples S6, S9, and S10. The isotherms shown in Fig. 8a,c,e are type IV with residual loops refers to mesoporous materials. The specific surface area for mentioned samples is 30.98 m^2^ g^−1^, 55.264 m^2^ g^−1^, and 31.301 m^2^ g^−1^ respectively. The total pore volume for the samples is 0.058439 cm^3^ g^−1^, 0.091263 cm^3^ g^−1^, and 0.083407 cm^3^ g^−1^ and the average diameter of the pores is 7.5454 nm, 6.6057 nm, and 10.659 nm, respectively.

a_s_,BET : S9 > S10 > S6.

Total pore volume(p/p0 = 0.927): S9 > S10 > S6.

Mean pore diameter: S10 > S6 > S9.

The amount of surface area and the distribution of holes of the samples are suitable, which indicates the optimal performance of the photocatalytic activity. Sample S9 has the highest specific surface area and the largest total pore volume and the lowest average pore diameter. This issue can increase the efficiency of photocatalytic activity among other samples. Figure 8g and h correspond to sample S7. The specific surface area for this sample is 50.311 m^2^ g^−1^, the total pore volume is 0.039783 cm^3^ g^−1^, and the average pore diameter is 3.163 nm. The surface area of this nanocomposite is close to sample S9. However, the ternary composite (S9) has a higher total pore volume with a larger pore diameter. According to these results, samples 9 and 7 are expected to provide higher photocatalytic efficiency because the number of their active sites is more.

### DRS analysis

**Figure 9:**
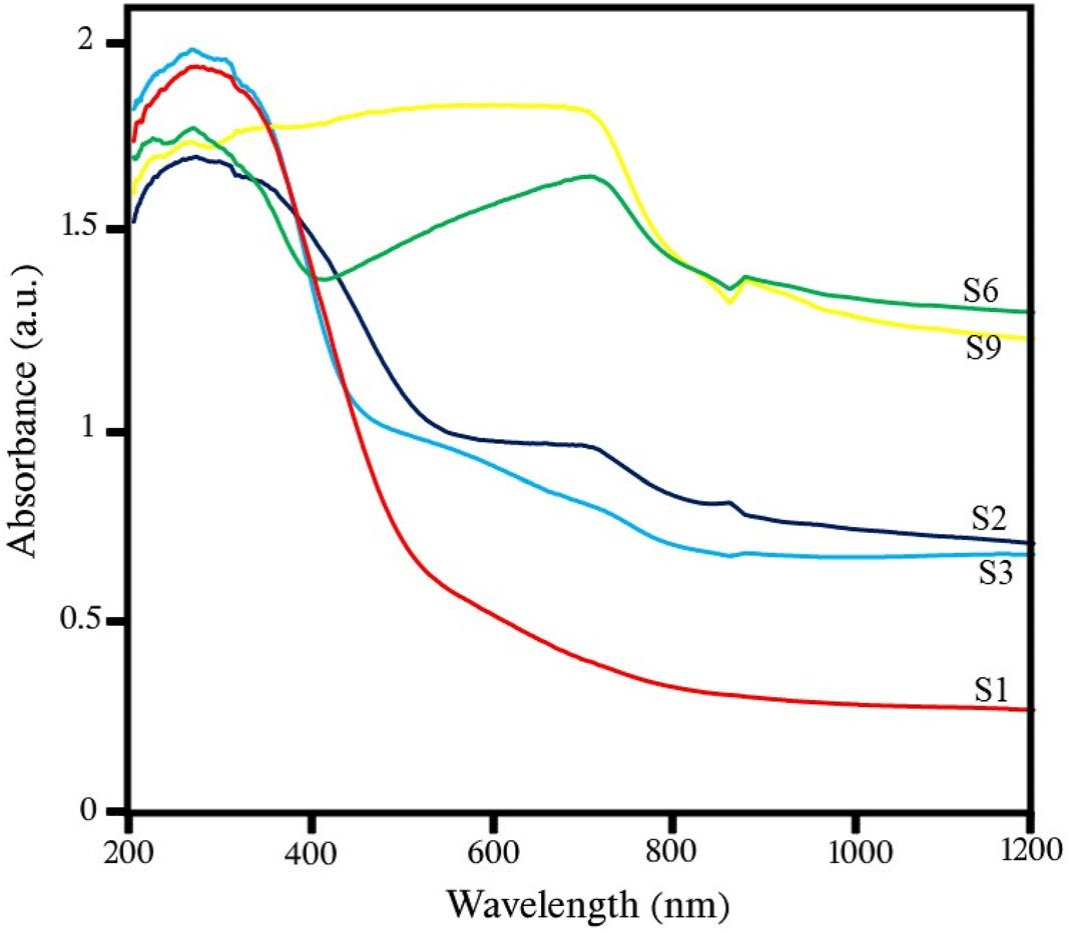
Absorbance spectra of the as-synthesized products.

Figure 9 shows the absorption graph of samples S1 (CeVO_4_), S2 (BiVO_4_), S3 (CeVO_4_/BiVO_4_), S6 (Ce_0.5_Bi_0.5_VO_4_/rGO), and S9 (Ce_0.5_Bi_0.5_VO_4_/rGO). Comparing samples S1–S3, which are without graphene, with samples S6 and S9, which contain graphene, the impact of graphene on the absorption rate is revealed. Samples containing graphene have good absorption in both ultraviolet and especially visible regions. By examining and comparing the spectrum of sample S3 with samples S1 and S2, it is clear that by adding CeVO_4_ to BiVO_4_, the amount of absorption in the ultraviolet region is improved, which can increase the efficiency of photocatalytic desulfurization. Samples S6 and S9, which contain Ce_0.5_Bi_0.5_VO_4_/rGO, have significant absorption in both the visible and ultraviolet regions. Sample S9 was synthesized under temperature control conditions and has the highest light absorption rate. Therefore, the highest photocatalytic desulfurization efficiency is expected to be related to Ce_0.5_Bi_0.5_VO_4_/rGO nanocomposite.

### The results of photocatalytic desulfurization of benzothiophene

**Figure 10:**
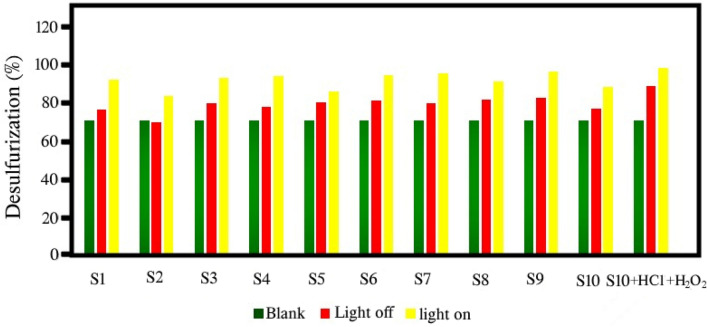
Percentage of photocatalytic and oxidative desulfurization of the as-synthesized products.

**Figure 11:**
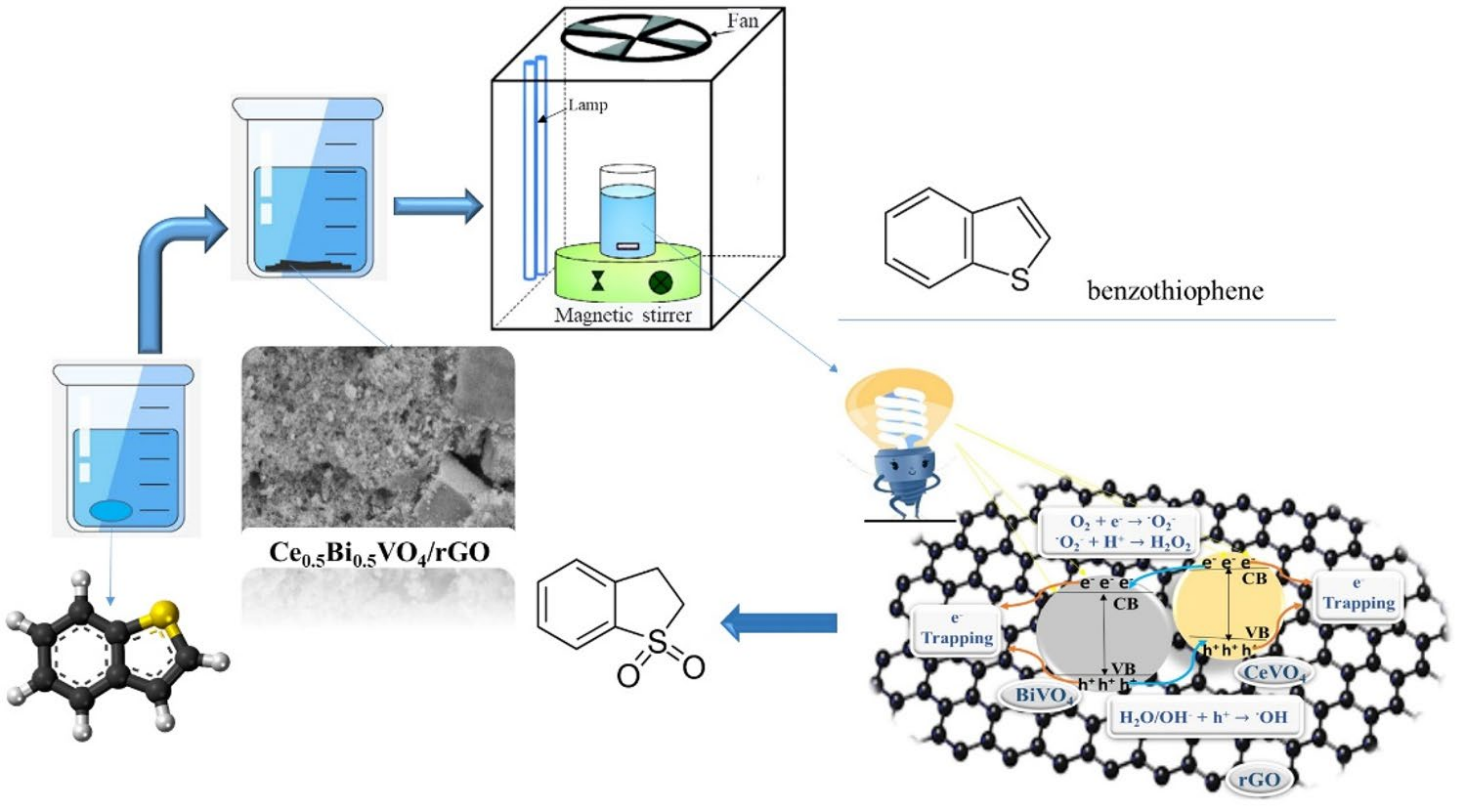
Schematic for the photocatalytic oxidation mechanism of benzothiophene by Ce_0.5_Bi_0.5_VO_4_/rGO nanocomposite.

Figure 10 and Table 2 present the results of photocatalytic oxidative desulfurization (PODS) of benzothiophene by the synthesized products. The green graph shows the amount of desulfurization in the absence of light and photocatalyst (only the oxidation reaction is performed) and as can be seen, the amount of thiophene desulfurization is 70.17%. This amount has reached ~ 95% in the presence of light and photocatalyst, where both the oxidation process and the photocatalyst process are carried out. The highest amount of desulfurization is related to sample S9 with 96.38% desulfurization efficiency. As revealed from the DRS results, this sample has a higher absorption, which is one of the reasons for the higher photocatalytic efficiency of this sample compared to other samples. As it is clear from the results, three-component nanocomposites have performed better than two-component nanocomposites in this process. Samples S1–S3 have lower efficiency than samples S4–S6 and this result shows that the combination of these materials and making nanocomposite along with rGO has increased the photocatalytic properties. Reduced graphene oxide (rGO) can help trapping electron transfer to form π–π electrons^25^. Figure 11 shows a schematic of the photocatalytic desulfurization mechanism performed by CeVO_4_/BiVO_4_/rGO. A reliable pathway for photocatalytic desulfurization can be given by the following equations:

4 $$ {\text{CeVO}}_{{4}} {\text{/BiVO}}_{{4}} /{\text{rGO }} + {\text{ h}}\upnu \, \to {\text{ CeVO}}_{{4}}{^{ * }} {\text{/BiVO}}_{{4}}{^{ * }} {\text{/rGO }} + {\text{ e}}^{ - } + {\text{ h}}^{ + } $$

5 $$ {\text{e}}^{ - } + {\text{ O}}_{{2}} \to {}^{ \cdot }{\text{O}}^{ - }{_{{2}} }$$

6 $$ {\text{O}}^{ - }{_{{2}}} + {\text{ 2H}}^{ + } \to {\text{ H}}_{{2}} {\text{O}}_{{2}} $$

7 $$ {\text{h}}^{ + } + {\text{ OH}}^{ - } /{\text{H}}_{{2}} {\text{O }} \to {}^{ \cdot }{\text{OH}} $$

8 $$ {\text{BT }} + {}^{ \cdot }{\text{O}}^{ - }{_{{2}}} /{\text{H}}_{{2}} {\text{O}}_{{2}} /\cdot  {\text{OH}} \to {\text{Oxidized BT}} $$

**Table 2:** Desulfurization rate of the as-synthesized products.

Sample no	30 min dark + 0 min light	30 min dark + 10 min light	30 min dark + 20 min light	30 min dark + 40 min light
S1 (CeVO_4_)	76.77	78.61	84.75	92.27
S2 (BiVO_4_)	70	77.51	82.75	83.75
S3 (Ce_0.5_Bi_0.5_VO_4_)	79.83	89.12	93	93.37
S4 (CeVO_4_/rGO)	78.24	80.07	86.37	94
S5 (BiVO_4_/rGO)	80.44	81.05	84.12	86
S6 (Ce_0.5_Bi_0.5_VO_4_/rGO)	81.42	90.71	94.62	94.75
S7 (CeVO_4_/rGO)	79.83	81.66	88	95.62
S8 (BiVO_4_/rGO)	82.03	83.37	89.25	91.25
S9 (Ce_0.5_Bi_0.5_VO_4_/rGO)	83.01	92.3	96.25	96.38
S10 (Ce_0.5_Bi_0.5_VO_4_/rGO)	77.02	77.38	80.37	88.75
S10 + H_2_O_2_ + HCl	89	91.56	91.81	98.65
Blank	69.95	71	71	71.17

**Figure 12:**
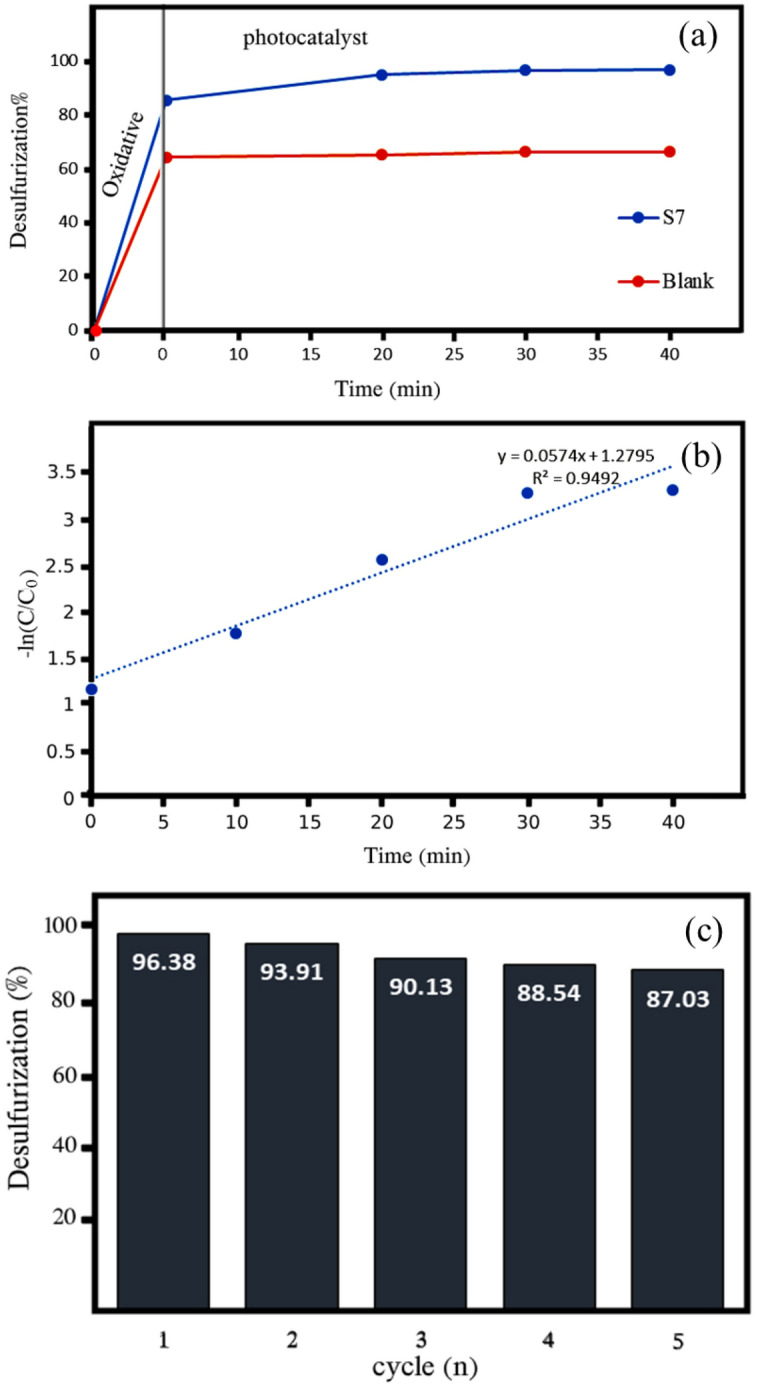
(**a**) Diagram of photocatalytic activity, (**b**) the reaction kinetics of photocatalytic desulfurization, and (**c**) the photocatalytic desulfurization recyclability of Ce_0.5_Bi_0.5_VO_4_/rGO nanocomposite (sample S9) for 5 cycles.

Samples S7–S9 have higher photocatalytic properties than samples S4–S6, which shows the effect of nanocomposite particle size on photocatalytic activity. In these samples, with temperature control, the dispersion distribution of particle size was lower and the particles were smaller and more uniform. The sample S10 has a lower photocatalytic efficiency than the sample S9, which indicates the effects of morphology on the photocatalytic process. In sample S10, bismuth vanadate and cerium vanadate are accumulated on the surface of graphene, while in other samples, the accumulation of cerium vanadate and graphene is on the surface of bismuth vanadate. The efficiency of the sample S6 is lower than that of the sample S10, since the temperature is not controlled in the synthesis process of sample S6 and therefore the increase in temperature has caused the growth of particles in this sample, which has reduced the photocatalytic property. When HCl and H_2_O_2_ were added, the efficiency of photocatalytic desulfurization by sample 10 increased and reached 98.66%. The use of HCl and H_2_O_2_ and their amounts were according to the results of the previous work done by Shahbazkhani et al.^36^. Therefore, by adding HCl and H_2_O_2_, almost complete desulfurization can be done in less time using synthesized products, especially sample S9. In order to determine the amount of oxidation and photocatalytic capability, the desulfurization graph of S9 and blank sample is shown in Fig. 12a. As shown, the rate of oxidative desulfurization in this study is about 70%. Time zero is where the rate of photocatalytic desulfurization is zero and only oxidative desulfurization has taken place. Therefore, it can be supposed that the synthesized photocatalyst (sample S9) increases the efficiency from 70.17 to 96.38%, which can be a significant amount. Figure 12b shows the reaction kinetics of photocatalytic desulfurization of benzothiophene over Ce_0.5_Bi_0.5_VO_4_/rGO (sample S9) nanocomposite. The plot of −Ln(C/C_0_) versus time shows straight line with the rate constant of 5.74 × 10^−2^ min^−1^. It is clear that there is a linear relationship between Ln(C/C_0_) value and the irradiation time, where C is the sulfur concentration at irradiation time t, and C_0_ is the sulfur concentration before irradiation and after the adsorption/desorption equilibrium. Good correlations show that the reaction kinetics follow a first-order rate law. Figure 12c relates to the recyclability of the synthesized nanocomposite in the photocatalytic process. According to Fig. 12c, after 5 reuses, only a slight decrease in desulfurization efficiency was observed, which indicates the high capability of this nanocomposite.

Compared to previous works reported for desulfurization of thiophene compounds^17,18,36^, our work has a higher efficiency. In our work, CeVO_4_/BiVO_4_/rGO nanocomposite was used for desulfurization for the first time. By using Ce_0.5_Bi_0.5_VO_4_/rGO nanocomposite under ultraviolet light, the efficiency of more than 96% was obtained in 40 min, which is a favorable result. Also, CeVO_4_/BiVO_4_/rGO was synthesized by sonochemical method for the first time. Using hydrazine, graphene oxide (GO) was reduced to rGO, and as a result, the one-step synthesis of the aforementioned nanocomposite was realized. Compared to the previous work reported for the one-step synthesis of CeVO_4_/BiVO_4_/rGO nanocomposite that used ethylene glycol solvent and solvothermal method^24^, our work is easier and more economical.

## Conclusions

In this study, Ce_0.5_Bi_0.5_VO_4_/rGO nanocomposite was synthesized by sonochemical method once without temperature control and once with temperature control between 0 and 5 °C. The products were identified using XRD, FT-IR, EDS, FESEM, BET, and DRS analyzes and were used for photocatalytic oxidative desulfurization. The desulfurization efficiency of CeVO_4_/rGO, BiVO_4_/rGO, and Ce_0.5_Bi_0.5_VO_4_/rGO nanocomposites synthesized in the absence of temperature control was 94, 86, and 94.75%, respectively, while with temperature control, the efficiency increased to 95.62, 91.25, and 96.38%, respectively. By adding CeVO_4_ to BiVO_4_ to form a Ce_0.5_Bi_0.5_VO_4_/rGO composite, the efficiency of desulfurization increases due to the increase of light absorption and decrease of charge recombination rate. By comparing the synthesized nanostructures without rGO and the nanocomposites containing rGO, it was found that rGO significantly increases the desulfurization efficiency. In addition to increasing the amount of light absorption and active sites, graphene also reduces the rate of electron–hole recombination. The desulfurization efficiency of the synthesized Ce_0.5_Bi_0.5_VO_4_/rGO was 88.75% in the absence of ultrasonic waves, which increased to 98.65% after the addition of HCl and H_2_O_2_. Comparing the efficiency of the oxidation process with the efficiency of the photocatalyst process revealed that the synthesized photocatalyst can increase the efficiency from 70.17 to 96.38%, which is a significant increase.

## Data Availability

The datasets used and analyzed during the current study available from the corresponding author on reasonable request.
